# The buzz around biodiversity decline: Detecting pollinator shifts using a systematic review

**DOI:** 10.1016/j.isci.2023.108101

**Published:** 2023-09-30

**Authors:** Sarah Whipple, Gillian Bowser

**Affiliations:** 1 Graduate Degree Program in Ecology, Colorado State University, 102 Johnson Hall, Fort Collins, CO 80523-1021, USA; 2 Department of Ecosystem Science and Sustainability, Colorado State University, Fort Collins, CO 80523-1021, USA

**Keywords:** Ecology, Biological sciences, Zoology, Entomology

## Abstract

Climate and land use change are two of the largest drivers of worldwide biodiversity loss, but detecting drivers of insect decline is more complex. Online data sources can elucidate such responses while identifying systematic data gaps. Using a systematic review, we found 119 studies that document bumble bee and butterfly responses to climate change. While bee literature was limited, there is high confidence that species are emerging earlier (∼17 days), mismatching with floral resources (100% of studies), and changing range distributions (−25%). More butterfly literature was available but did not yield consistent responses. Evidence shows earlier emergences (∼5 days), decreasing range distributions (−19%), and population shifts amongst generalist (87% increase) versus specialist (65% decrease) groups. We argue that the effect of changing climates on floral emergence, abundance, and distribution may be more significant than the impact of climate change on biodiversity; however, further research is required, particularly within the Southern Hemisphere.

## Highlights

• Insect pollinator groups face worldwide decline due to climate and land use change
• We synthesized bumble bee and butterfly species responses to climate change
• Bumble bees are emerging earlier, mismatching resources, and contracting in range
• Butterflies are emerging earlier but are inconsistent in other response patterns

## Graphical abstract

**Figure:**
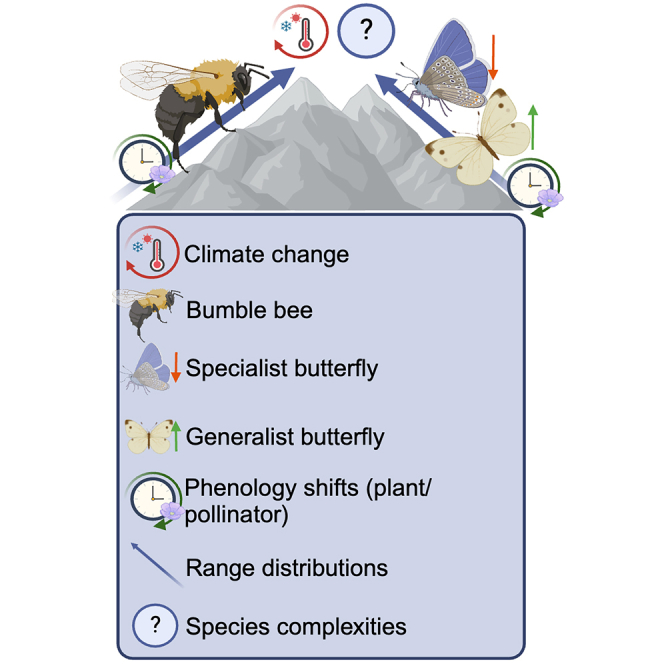

## Introduction

Climate change is emerging as the most significant anthropogenic driver of biodiversity loss; however, the rate at which different groups of species change in response to these drivers is surprisingly variable at the regional level, as well as differs widely among species.^1^^,^^2^^,^^3^^,^^4^^,^^5^ Yet, under the business-*as*-usual emissions scenario at the Representative Concentration Pathway (RCP) 8.5, 81% of terrestrial species assemblages are expected to be exposed to unprecedented warming before 2100, which will have detrimental effects on species persistence to the point of irreversible tipping points of survival and system collapse.^6^^,^^7^

The impact of temperature warming, changing precipitation levels, and a decrease in snowpack has mixed effects on pollinator species across the world.^1^ Some species have been able to adapt and shift their range or establish in new areas as spring and summer seasons lengthened, whereas many species have risked population declines due to impending pressures such as habitat loss, decreased thermal tolerances, and resource competition.^8^^,^^9^^,^^10^^,^^11^^,^^12^^,^^13^ Pollinator species are sensitive to temperature and precipitation fluctuations. For bumble bees, climate thermal limits can exceed the physiological thermal limits of the species, leading to range shifts or declines in species richness.^11^ Butterflies, equally dependent on temperature to break diapause and for nectar and larval resources, can be sensitive to increasing temperatures and drought conditions, which, in turn, can have negative effects on populations.^14^ Additionally, pollinators can adapt to challenges such as climate anomalies, but the persistence of species, especially those that are resident to the area or have limited elevational ranges, face uncertainty as ecosystem disturbances increase in severity.^15^^,^^16^^,^^17^

The Intergovernmental Panel on Climate Change (IPCC) projects that 10–40% of mean snowpack worldwide will decrease from 2031 to 2050, and additionally, if following the RCP 8.5 scenario, snowpack will decrease 50–90%.^6^ Specific climate change variables, such as temperature, are not always a direct cause of a decline in insect pollinators that rely on floral resources for survival and as host plants, yet the resultant effect of changing climates on floral emergence and abundance may be more significant than the impact of climate change on pollinator presence itself.^4^^,^^15^^,^^17^^,^^18^ For pollinator species dependent on early emerging spring flowers, earlier trends of snowpack decline from a seasonal perspective will shift floral phenological patterns and restrict floral resource availability.^18^^,^^19^^,^^20^^,^^21^

While the impacts of warming may have a mixed response on the persistence of plant and insect species worldwide, the certainty of individual species’ reactions to changes in climate are relatively unknown. Species can be preserved through conservation and adaptation measures on a global scale, as well as with growing networks of protected areas focused on preserving critical ecosystem services like pollination.^6^ However, more information is needed on the dynamics between climate change and pollinator species resilience, species’ responses to future climate variation, and the impacts of anthropogenic disturbances on pollinator species diversity over time.^21^^,^^22^^,^^23^^,^^24^ Researchers need to better understand those species who will be most vulnerable to climate change, as well as those that may thrive under altered land and climate scenarios.^22^ Because international organizations such as the IPCC know that changes to ecosystems due to anthropogenic forces have affected seasonality, species distribution, and overall ecosystem functioning, it is critical to document current changes happening, relate these effects back to historical species distributions, and project trends.^25^ This will help in determining species resilience toward changing climates,^26^ as well as identify areas where stronger conservation measures may need to occur to prevent irreversible feedback loops.^27^

Due to these uncertainties present in the literature on pollinators and climate change, this research consolidates previous studies on bumble bee and butterfly responses to climate variation to inform researchers on taxon sensitivities to changing landscapes. The causes and rate of pollinator decline tied to climate change are undefined, and this research specifies such responses and the agreement of such patterns that are found within the literature. We used a systematic review approach by compiling previous studies with similar data metrics to analyze the effect of changing climates on bumble bee and butterfly species responses. Bumble bees and butterflies acted as the target taxa for this review given their international attention in conservation efforts, ease in accurate taxonomic identifications, as well as their known sensitivities to changing climates.^1^ Additionally, the geographic and historic breadth of taxonomic understanding for both bumble bee and butterfly species surpasses other native bee and some moth groups, which allowed for analyses across broader spatial and climate change scales.^28^^,^^29^ Regardless, species response patterns found through this synthesis are expected to translate toward taxonomically similar species groups.

## Results

### Systematic review trends

**Figure 1:**
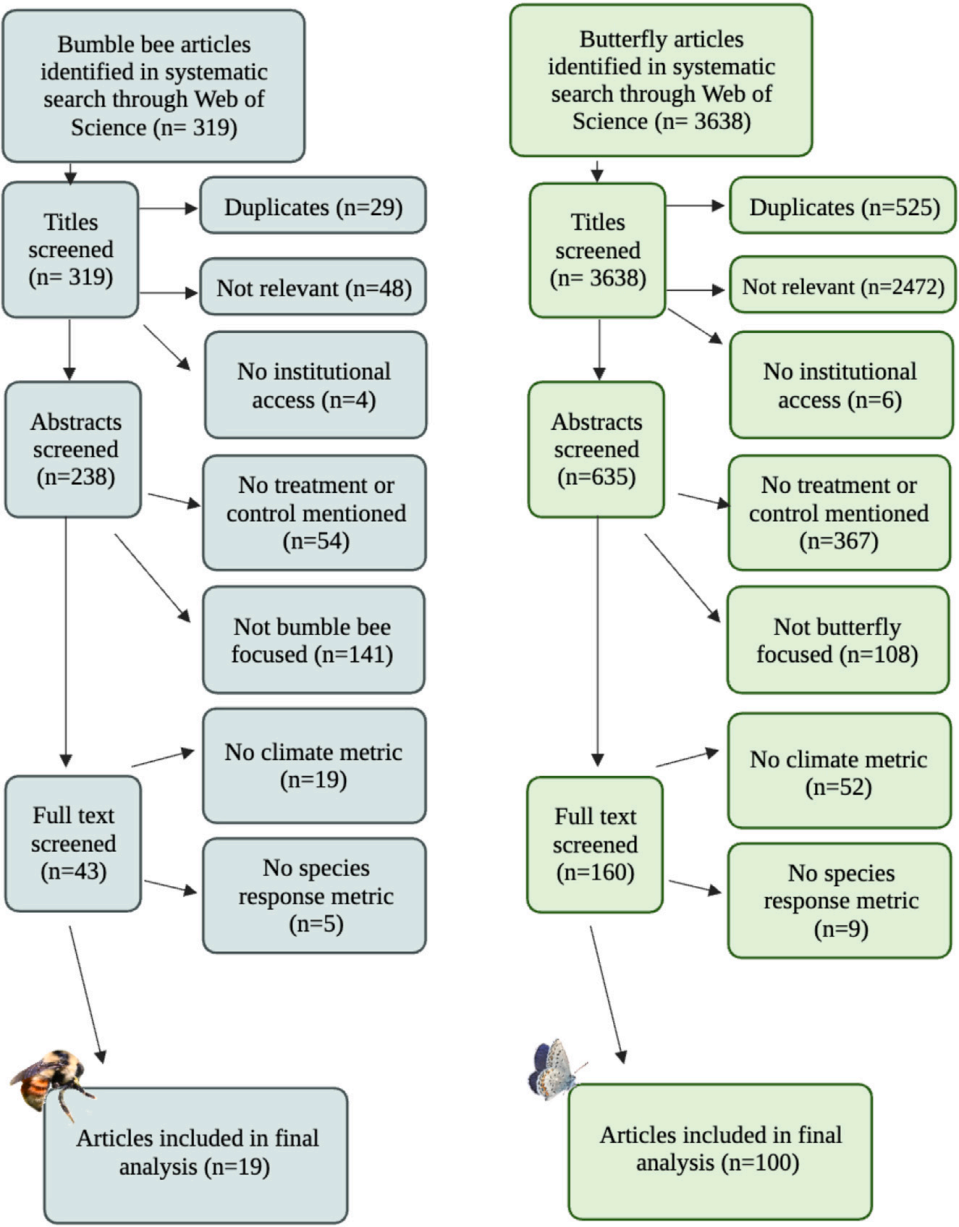
Systematic Review Flow Chart, related to the STAR method details Section Flow chart identifying relevant literature for the species-climate response systematic review. Based on data metrics, 19 bumble bee and 100 butterfly papers were used for the analysis. Figure created with BioRender.com.

Initial Web of Science results yielded 319 bumble bee and 3,638 butterfly articles. After refining articles based on relevancy, institutional access, and experimental metrics, 19 bumble bee and 100 butterfly articles were used in this review (Figure 1). Bumble bee trends came from 60 countries and seven US states, while butterfly trends came from 61 countries and 30 US states. There was a predominant focus on taxon-wide studies across both bumble bee (15 taxon-wide; four species-specific studies) and butterfly (71 taxon-wide; 29 species-specific studies) literature. The exceptions included a few articles that highlighted species known to be rare or in decline (i.e., *Danaus plexippus*, monarch; *Speyeria idalia*, regal fritillary; *Bombus funebris*, gray-backed bumble bee), or the dominant/charismatic species observed within an ecosystem (*Parnassius apollo*, Mountain Apollo; *Pieris rapae*, Cabbage White). For bumble bees, the most common climate variables studied included temperature (53% of articles), snowmelt date (26%), fire (21%), and precipitation (21%). For butterflies, the most common climate variables studied included temperature (58% of articles), precipitation (35%), and fire (16%). Some articles overlapped in study taxa, with three publications focusing on Lepidoptera (butterflies and moths) and bee-Hymenoptera genera (including bumble bees), and six publications focusing on butterflies and moths. However, only bumble bee and butterfly reported data were included in the following results. For article information, including study taxa and location, see Tables S1 and S2.

### Evidence and agreement trends

The evidence and agreement statements for each species response to climate variables are reported in Tables 1 and 2. These statements follow the confidence level evidence and agreement statements used by the IPCC and IPBES.^30^ For bumble bee studies, earlier plant phenology, generational or life cycle emergence changes, and overall species emergence shifts were responses with medium- (70–89% agreement) to high- (>90% agreement) confidence, but with limited evidence available (<10 publications) (Table 1). Butterfly literature observed medium-to high-confidence of earlier trends in categories such as species emergence and plant phenology emergence patterns, and with medium- (10–30 publications) to limited-evidence. Changes in univoltine (single generation producing species) and multivoltine (multiple generation producing) species generations indicated earlier, additional generation occurrences; however, this response on life history strategies had limited evidence available.

**Table 1:** Species phenology and life stage responses related to the STAR quantification and statistical analysis section

	Phenology/Species Emergence Trends	Species Emergence	Plant Phenology/Emergence (Phenological Mismatch)	Generations/Life Cycles
*Bumble Bees*	Late	40%	0%	0%
	Early	60%$$	100%∗∗	100%∗∗
	Uncertain effect	0%	0%	0%
	Total Publications	5	6	1
*Butterflies*	Late	36%	0%	40%
	Early	*64%$$*	100%∗∗	60%$$
	Uncertain effect	0%	0%	0%
	Total Publications	22	1	5

Evidence and agreement statistics indicating phenology and life stage species responses to climate variables for papers used in the systematic review (n = 19 papers for bumble bees and n = 100 for butterflies). For these responses, there were five bumble bee and 22 butterfly species emergence publications, six bumble bee and one butterfly plant phenology publications, and one bumble bee and five butterfly life cycle publications. Stars (∗∗) indicate high confidence (>90%), pound symbols (##) indicate medium confidence (70–89%), dollar signs ($$) indicate low confidence (50–69%) and no additional symbol () indicates very low confidence (<50%) in the species response. Italicized percentages indicate medium evidence (10–30 publications); underlined percentages indicate limited evidence (<10 publications).

**Table 2:** Species population and distribution responses related to the STAR quantification and statistical analysis Section

		Response	Richness	Abundance	Population Change	Specialist Change	Generalist Change	Range
*Bumble Bees*	Population Responses	Increase Effect	40%	89%##	50%$$			
		Decrease Effect	60%$$	11%	50%$$			
		Uncertain Effect	0%	0%	0%			
	Distribution	Expansion						0%
		Contraction						100%∗∗
		No Change						0%
	Total Publications		5	9	2			5
*Butterflies*	Population Responses	Increase Effect	*59%$$*	36%	*67%$$*	29%	*87%##*	
		Decrease Effect	*35%*	*60%$$*	*58%$$*	*65%$$*	13%	
		Uncertain Effect	6%	4%	4%	6%	0%	
	Distribution	Expansion						*56%$$*
		Contraction						*41%*
		No Change						3%
	Total Publications		34	25	15	17	15	32

Evidence and agreement statistics indicating population and distribution species responses to climate variables for papers used in the systematic review (n = 19 papers for bumble bees and n = 100 for butterflies). For these responses, there were five bumble bee and 34 butterfly richness publications, nine bumble bee and 25 butterfly abundance publications, two bumble bee and 26 butterfly population change publications, 17 and 15 butterfly specialist and generalist publications, and five bumble bee and 32 range/altitudinal change publications. Stars (∗∗) indicate high confidence (>90%), pound symbols (##) indicate medium confidence (70–89%), dollar signs ($$) indicate low confidence (50–69%) and no additional symbol () indicates very low confidence (<50%) in the species response. Italicized percentages indicate medium evidence (10–30 publications); underlined percentages indicate limited evidence (<10 publications).

Within the population and distribution species responses (Table 2), bumble bees trended toward decreases in richness but increases in species abundance; both responses were considered low and medium confidence (50–89% agreement) and had limited evidence (<10 publications). Bumble bee range contractions and overall population changes in response to climate variables were low confidence findings, with 50% of studies indicating increases in populations, and 100% of studies finding decreases in species range distribution (contractions). Butterflies had mixed responses in species richness and abundance trends, with nearly all trends having <50% agreement, or low confidence. The most prominent responses included decreases in species abundance (medium evidence) and increases in species generalists’ (medium evidence, increase of 87%). Compared to bumble bees, only 41% of butterfly studies measuring changes in range distributions have observed species range contractions.

### Species response trends

**Figure 2:**
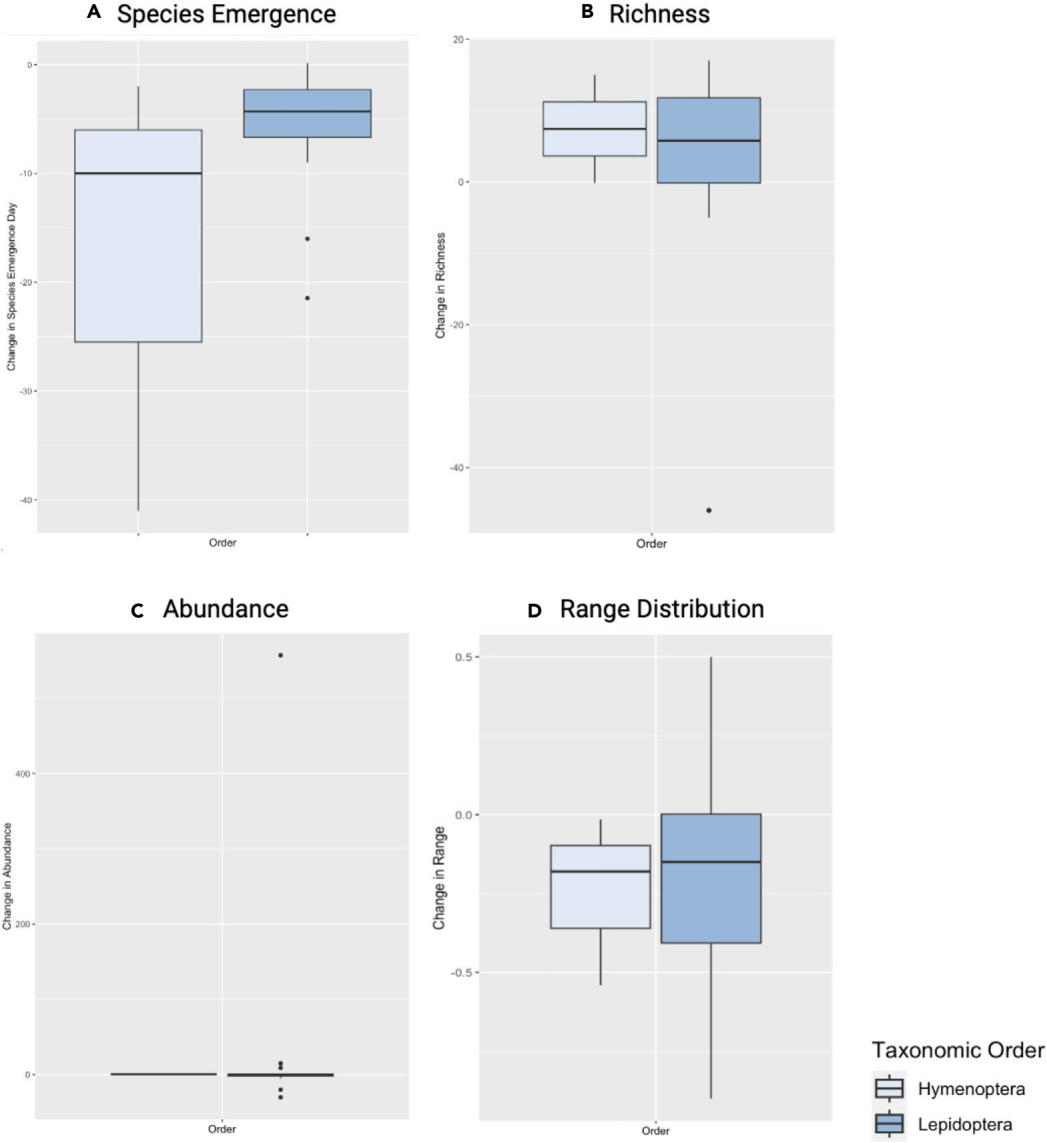
Bumble Bee and Butterfly Species Response T-Test Distribution related to the STAR quantification and statistical analysis Section (A–D) Bumble bee (order Hymenoptera) and butterfly (order Lepidoptera) species responses, including change in emergence day (A), change in richness (B), change in abundance (C), and change in range distribution (D). Bumble bee responses (light blue) were more similar, except for within the emergence category (A). Butterfly responses (dark blue) were more spread out, especially within the abundance category (C), where strong outliers occurred.

Using the species response metrics with the most available data, the publications with relevant data included nine bumble bee and 45 butterfly studies. These studies included field collections, long-term monitoring datasets, and species modeling exercises that analyzed the effect of temperature change on historic species responses. Given data availability, changes in species emergence, richness, abundance, and range distribution were the four responses compared amongst bumble bees and butterflies (Table 3; Figure 2). Both bumble bees and butterflies have observed changes in emergence days, with bumble bees emerging on average 17.67 days earlier and butterflies emerging 5.76 days earlier (Figure 2A); the butterfly emergence changes observed amongst publications were considered significant (p < 0.001). The average bumble bee (7.43) and butterfly (2.39) population richness in response to temperature change has increased (Figure 2B), as well as abundance (0.68 bumble bee; 34.97 butterfly) (Figure 2C). The changes in range distribution also varied between species; bumble bees have seen range contractions of nearly 25%, while butterflies have seen range contraction of 19% (Figure 2D).

**Table 3:** Species response (Emergence, Richness, Abundance, and Distribution) T-tests related to the STAR quantification and statistical analysis section

	# Studies	t	df	p value	Lower 95% CI	Upper 95% CI	Mean
**Species Emergence (Days Early (−)/Late (+))**							

*Bumble Bee*	3	−1.48	2	0.28	−68.84	33.50	−17.67
*Butterfly*	16	−4.02	15	0.001∗	−8.82	−2.70	−5.76

**Richness (# Unique Species)**							

*Bumble Bee*	2	0.98	1	0.51	−88.82	103.67	7.43
*Butterfly*	14	0.58	13	0.57	−6.56	11.33	2.39

**Abundance (Total # Species)**							

*Bumble Bee*	3	1.74	2	0.22	−1.00	2.37	0.68
*Butterfly*	15	0.94	14	0.37	−45.22	115.16	34.97

**Changes in Species Distribution Range (%)**							

*Bumble Bee*	3	−1.58	2	0.25	−0.91	0.42	−0.25
*Butterfly*	13	−1.75	12	0.10	−0.43	0.05	−0.19

One-tailed unequal variance t-tests for bumble bee and butterfly responses. p-values <0.05 denoted with ∗ and are considered significant. Butterfly species emergence responses were significant.

### PCA trends

**Figure 3:**
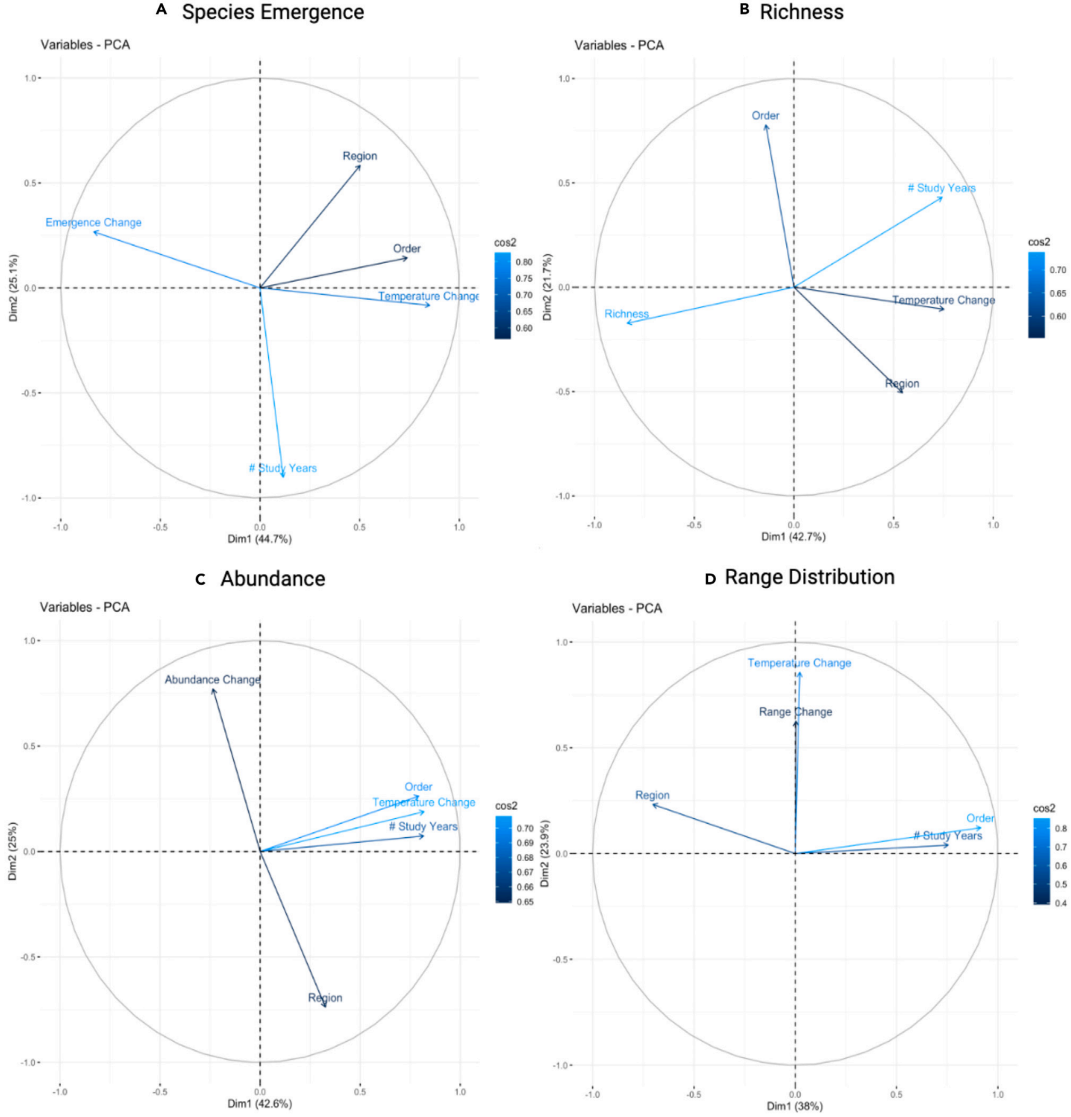
Bumble Bee and Butterfly Species Response Principal Component Analyses related to the STAR quantification and statistical analysis Section (A–D) PCA correlation plot representing bumble bee and butterfly change in emergence (A), change in richness (B), change in abundance (C), and change in range distribution (D) responses in relation to temperature variation. Positively correlated variables are grouped together, whereas negatively correlated variables oppose each other along the plot origin (ex: region and abundance change). Arrows further away from the origin represent strong variable representation; those closer to the origin represent weak variable representation.

The principal component analysis (PCA), a multivariate statistical tool used to simplify multidimensional datasets, collated the 54 studies mentioned above, which focused on temperature changes and their response on species emergence, richness, abundance, and range distribution. PCA plots for bumble bee and butterfly trend data highlight significant correlations between species responses and temperature variation (Figure 3). Within the species emergence dataset, temperature change and species groups (bumble bees versus butterflies) had strong, negative correlations with one another; on the other hand, species emergence changes and study regions had no correlation (Figure 3A). All species emergence variables, except for study region and taxonomic order, were well represented within the PCA, with quality of representation values above 75%. The species richness dataset had negative correlations between changes in richness and the number of study years, and between taxonomic order, region, and temperature changes (Figure 3B). Richness changes and study years had the strongest representation within the PCA, with both variables above 70%. The abundance dataset had negative correlations between regions and abundance changes, and positive correlations between temperature change, taxonomic order, and study years (Figure 3C). Within the distribution dataset, temperature change and range change were positively correlated, while the study region, taxonomic order, and study duration had no strong correlation (Figure 3D). Temperature change and taxonomic order had the highest quality of representation values, with both variables at 75%.

The PCAs validate the correlation of species responses to climate changes. For species emergence (Figure 3A), the negative correlation between temperature and emergence dates aligns with the rate of change occurring within species’ groups across regions; butterflies, on average, have emerged ∼12 days later than bumble bees, yet both are emerging at least one week earlier than previously observed. Study regions were also important to consider, as regional variation had positive correlations with taxonomic groups amongst emergence studies and negative correlations with richness and abundance studies (Figures 3B and 3C). The evidence and agreement analyses found general decreases in bumble bee richness, but this was not the trend for temperature/richness studies. This is reemphasized through the t-tests and boxplots, specifically for richness (Figure 2B) and abundance (Figure 2C). Studies that observed strong increases in bumble bee abundances and decreases in butterfly richness and abundance provide region-specific and species-specific (rather than taxa-specific) examples where few taxa have benefited from changing climate conditions. Additionally, all response category PCAs indicate that temperature acts as a strong representative variable; however, temperature change was only negatively correlated with the emergence “response”, and instead had correlations to other variables such as taxa and region. This presents the importance of species context, in the form of biodiversity and habitat/regional diversity, and how landscape and community resilience may be more revealing of climate change effects on species responses.

## Discussion

This systematic review regarding pollinator species responses to climate changes demonstrates that generalizing species responses, especially for short-lived groups such as invertebrates, is complex. Species life history traits, phenology, distribution, and population patterns over time all interact with climate variables such as temperature. This has led to different responses such as range shifts or contractions, population increases or declines. While there is general agreement on the overall decline in insect pollinator populations and biodiversity loss associated with changing climate conditions, the effect of such changes on pollinator populations is more variable.^24^ As highly mobile species with the potential to migrate in response to warming temperatures, changing habitats, and floral resource availability, bumble bees and butterflies offer opportunities to ascertain species’ responses to unprecedented conditions and use these responses as indicators of ecosystem resilience. However, all these results should be taken with some level of uncertainty given sample sizes with strong taxonomic and geographic biases that emphasize the need for additional research in this field.

The evidence and agreement statements provide one approach to parsing species responses to different climate variables. Both bumble bee and butterfly groups show evidence of changing species emergence and phenological cues, as well as evidence of potential phenological mismatch through the desynchrony between plant and pollinator emergence periods. These ranged from continental studies to individual states, but do not paint a representative picture of global pollinator trends. Previously, literature focusing on phenological mismatch prioritized bee taxa,^13^^,^^31^ and this study affirms that changes may be more widespread across pollinator groups. Incorporating phenology/climate datasets into future pollinator studies will strengthen the understanding of species responses. The PCAs for pollinator responses to temperature change highlighted the importance of temperature to species population responses such as emergence, range distribution, richness, and abundance changes. The intensity of climate variation, as seen through temperature changes, had strong variable representation and correlation but not necessarily toward the response variables studied within the PCAs. Lastly, the distribution of bumble bees and butterflies have observed range contractions, with a few exceptions that fall into the non-native or resilient species category. While the cause of these range contractions may be less certain, whether due to direct warming temperatures, changing habitats, or species interactions, this finding does translate to pollinator populations observed worldwide.

### Visualizing and summarizing species trends

**Figure 4:**
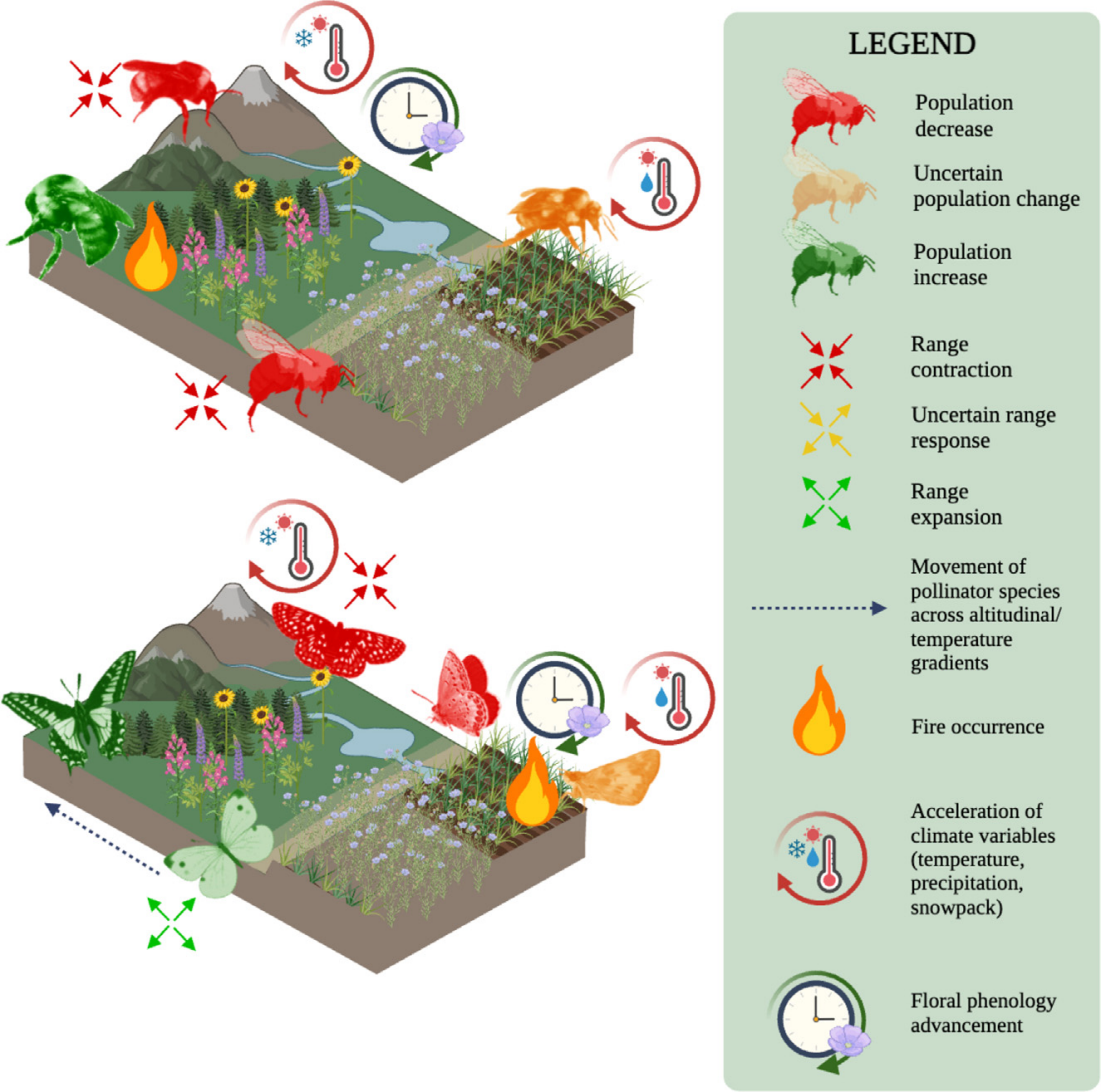
Bumble Bee and Butterfly Response Schematic to Climate and Land Use Variables related to the STAR quantification and statistical analysis Section Species schematics based on responses to climate variables observed in the literature. The species icons included do not necessarily represent species-specific patterns observed in the literature; rather, this figure serves as the general response of bumble bees and butterflies. Populations of bumble bees and butterflies found at higher altitudes and in areas with climate and land use changes faced greater rates of decline, while species capable of adapting to landscape pressures (i.e., generalists and non-native species) increased in population sizes. Some species had mixed, uncertain responses to climate variables. Figure created with BioRender.com.

Many species relationships observed declines in species richness, abundance, or range contractions in response to climate variables, and primarily in response to temperature change (Figure 4). Other climate responses were prevalent but did not have sufficient data to analyze further. The systematic review results found that butterfly univoltine species with earlier spring emergence patterns declined more consistently than those that could produce multiple generations (multivoltine) throughout the growing season, and this was related to a higher probability of climate extremes observed in the spring. These species responses speak to the resilience, or lack thereof, of pollinator species amidst a changing climate, and how their genetic, behavioral, and physiological functions may not be capable of adapting under future climate scenarios.

The uncertain population response category yielded results with stronger emphases on land use land cover change. Land use change is classified as shifts in habitat connectivity, degradation or modification, agricultural intensification, abandonment, or urbanization, and this driver has ample literature to argue its impact on pollinator decline.^32^^,^^33^^,^^34^^,^^35^ These studies argued that floral resource quality and quantity, as well as habitat conservation, would be more critical to maintaining species richness, abundance, emergence, and distributions, regardless of whether climate variables continue to change ecosystems in the future. They also justified increases in populations, either in the form of species richness, abundance, or range distribution, through land use changes that were beneficial to species, and particularly generalist, adaptable populations.

### Limitations of the study

One proposed driver of pollinator declines that was not highlighted in this review is the increased pervasiveness of non-resident and resilient species, which has shown to strongly impact pollinator richness, abundance, and health.^1^ Few papers examined the effects of non-native species, while many focused on single species or migratory species that could be negatively impacted by changing climate. The response of invasive species on pollinator persistence varies; species generalists may benefit from the introduction of new alien plant species, while species specialists may lose previous abundant floral resources or be outcompeted for resources due to the species invasion.^36^^,^^37^^,^^38^ Butterfly literature reported this response as well: the species specialist data trended toward declining populations, while the species generalist data trended toward increasing populations.^39^^,^^40^ Further climate/species studies should differentiate between resident and alien species in more depth to better understand the nuances between life histories, floral resource use and availability, and preferred habitat types of these species’ groups.

Additionally, the integration of other dynamic data resources, such as online datasets coming from Natural History Collections (NHCs), citizen science repositories, and other online sources could bolster the confidence of observed species trends.^41^ Because this study summarized findings from other publications, the limitations in data communicated across the literature meant that not all species responses could be analyzed through this method. Data biases present in the literature, such as spatial/geographic biases, literature language biases, concentration taxa, and the data findings (significant and non-significant) created limitations in plausible data analyses, such as using meta-analysis statistical techniques and providing a global understanding of species trends.^42^ Such limitations may diminish the significance of such trends; however, these biases are commonly observed problems across biodiversity/climate change studies that researchers across disciplines need to work to improve.^42^^,^^43^^,^^44^ As such, the inclusion of live, raw, and active datasets that incorporate real-time observations and effect sizes, and the mechanisms in which data are publicly available for use, should be considered in future biodiversity loss trend studies; this will help with future data comparisons for systematic reviews and meta-analyses. Data transparency through science publishing will ensure research dissemination is properly interpreted and maximized in its future impact.^45^ The integration of other datasets, such as floral phenology and landscape management changes, will also improve the understanding of species responses to landscape changes more directly impacted by climate change, and increase data availability at the international scale.^41^^,^^46^

Pollinators are facing multiple drivers of species decline, including climate change and, in some instances, land use change. While the strength of driving variables is at times uncertain, the trends of earlier species emergence, phenological mismatches, and range contractions are evident depending on the pollinator functional group of concern. However, the current literature can be used to detect important shifts in pollinator populations, and this systematic review acts as one example to better understand uncertain patterns of pollinator biodiversity loss due to climate change. Together, these results highlight the complexities of species responses, particularly in varying regions across the globe, as well as across species with different behavioral and life history patterns. Researchers can apply such findings toward individual insect taxa or broader pollinator functional groups to determine the effect of climate change on local, regional, and global biodiversity loss. Future studies, with emphases on broader spatial and taxonomic scales as well as widespread data dissemination with standardized data metrics, will only improve researchers’ understanding of climate change on pollinator biodiversity at the global scale. Worldwide collaboration on pollinator decline is urgently needed to reaffirm similar trends in lesser observed taxa and geographic areas. These findings, while dire in some instances, offer opportunities for additional species/climate studies, and provide taxonomic priorities for species conservation amongst a changing planet.

## STAR★Methods

### Key resources table

**Table:** 

REAGENT or RESOURCE	SOURCE	IDENTIFIER
**Deposited data**		

Mendeley Dataset, including data published/compiled from literature review	Web of Science/Mendeley Data	For compiled list, see Whipple, Sarah (2023), “Meta-analysis_Whipple_et_al_iScience2023”, Mendeley Data, V1, https://doi.org/10.17632/zj6hrgj5z4.1

**Software and algorithms**		

R Studio version 2023.06.1 + 524	Posit, PBC	https://posit.co/download/rstudio-desktop/

**Other**		

Web of Science	Clarivate	https://www-webofscience-com.ezproxy2.library.colostate.edu/wos/woscc/basic-search

### Resource availability

#### Lead contact

Further information and requests for resources should be directed to and will be fulfilled by the corresponding author, Sarah Whipple (sarahemmawhipple@gmail.com).

#### Materials availability

This study did not generate new unique materials.

#### Data and code availability

• Publication data have been deposited in Mendeley Data and are publicly available as of the date of publication.
• All original code for analyses has been deposited in Mendeley Data and is publicly available as of the date of publication.^47^ DOIs are listed in the key resources table.
• Any additional information required to reanalyze the data reported in this paper is available from the corresponding author upon request.

### Experimental model and study participant details

Through this study, we wanted to test the effect of climate change (i.e., temperature warming) on pollinator (bumble bee and butterfly) species responses. The independent variables included all climate variables studied across publications. This included temperature, precipitation, snowpack, fire, and other natural hazards, but we prioritized temperature change in our results given the data availability. The dependent variables included all pollinator responses studied across publications. This included species emergence, phenology, distribution, richness, and abundance. We prioritized richness, abundance, emergence, and distribution given the data availability. This experiment used no control variables, nor constants, to keep the largest subset of data available for the final analysis. Last, none of the studies used through this experiment were randomly assigned or manipulated; all studies occurred in natural environments, and the authors worked to assess a variety of search terms to minimize geographic and taxonomic biases present in literature queries. Study participants were not directly observed by the authors of this publication due to the systematic review framework, but all studies prioritized bumble bee and butterfly collections to answer their research questions.

### Method details

To collate all published articles with relevance to climate change impacts on bumble bees and butterflies, we used a variation of search terms in the Web of Science literature repository. These included the following: bumble bees AND climate change∗^48^; bumble bees AND global change∗^49^; bumble bees AND temperature warming∗^50^; bumble bees AND precipitation ∗^51^; bumble bees AND global warming∗^52^; bumble bees AND snowpack∗^53^; bumble bees AND fire∗^54^; butterflies AND climate change∗^55^; butterflies AND global change∗^56^; butterflies AND temperature warming∗^57^; butterflies AND precipitation ∗^58^; butterflies AND global warming∗^59^; butterflies AND snowpack∗^60^; butterflies AND fire∗.^61^ Boolean phrases ensured that results included research related to both search terms, and the asterisk ensured that words with different endings (such as fires, global changes, etc.) appeared as well.

The inclusion criteria for this systematic review followed the population, treatment, control, and outcome (PTRO/PICO) framework,^62^ a method used in systematic reviews and meta-analyses to identify papers with similar study groups, methods, and result metrics. The populations of concern included bumble bees (order Hymenoptera, genus *Bombus*) and the butterfly order (Lepidoptera) with special emphasis on butterfly species over moths. Both taxa were prioritized given their international attention in conservation literature.^1^ Even with this study parameter, populations of concern were studied worldwide but with strong data biases toward North American and European species responses, given the literature present and limited data available. Papers that reported results on taxa in addition to butterfly or bumble bee species, such as moths or additional bee genera, were not omitted from inclusion so long as specific bumble bee/butterfly data was reported. The treatments of relevance included field studies, historical data trend analyses, and theoretical modeling that incorporated field and/or historical datasets in their analysis. The overview literature dataset considered for this analysis required some combination of independent variable acting as a climate driver (i.e., temperature, precipitation, etc.), and its response on pollinators. Climate variables recorded from each publication reflected the change observed within the study location; for example, the temperature/precipitation value recorded was the change in an area observed over the study period. The outcomes of interest (dependent variable) included species responses to climate effects, either in the form of richness, abundance, emergence, or distribution patterns (direct responses to climate change) and/or evidence of plant-pollinator phenological responses (indirect responses to climate change). For butterflies, species generalists versus specialists and their responses to climate variables were also tracked. Life stage and generational trends were tracked for both bumble bees and butterflies. For bumble bees, the differentiation was made between queens and workers, and for butterflies, the difference was made between univoltine and multivoltine species. All mean reported data responses were recorded from each publication, then checked for consistencies with data reporting metrics. For example, distribution studies measured change in species range, either as a percentage or by meters of distributional change – the metric that was most consistent for that species response (percent change in range) was used in the analysis, while papers that did not follow the same consistent metrics were omitted from the final analysis. Studies that fit the treatments and outcomes criteria, including appropriate data metrics, were included in the final data analysis. Studies were omitted if they were not considered primary literature, such as reviews, theoretical models that did not incorporate historical/field-collected datasets, meta-analyses, editorials, or commentaries. Additionally, publications that were found as results within Web of Science but ended up having no institutional access from Colorado State University were omitted. For a full breakdown of the raw data collected from each publication, please refer to the publication’s Mendeley dataset, which includes publication details from the Web of Science query, overview information, statistics, and R code.^47^

### Quantification and statistical analysis

#### Systematic review data analysis

Each species response received an evidence and agreement statement to indicate the confidence level of certainty for the projected species response.^30^ Both variables followed the Intergovernmental Panel on Climate Change (IPCC) guidance for addressing scientific certainty in high-level research reviews. Results with greater than 90% species response agreement were considered “high confidence,” while those with 70–89% and 50–69% were considered “medium” and “low confidence.” Results with less than 50% agreeance were considered “very low confidence.” Evidence of more than 30 publications were considered “robust evidence,” while those between 10 and 30 publications were considered “medium evidence,” and fewer than 10 publications were considered “limited evidence.” For the evidence and agreement statement analyses, species response trends were generalized into “early”, “late”, and “no effect” for temporal responses, and “increase”, “decrease”, and “no effect” for population responses. All sample sizes for the evidence and agreement statements can be found in Tables 1 and 2.

Based on the evidence and agreement results, data from both bumble bee and butterfly studies were then grouped into publications measuring the same species responses and utilized the same data metrics. These publications all assessed the effect of temperature change on species, i.e., all other climate variables were eliminated from the analysis to maintain data consistencies. Then, to test if bumble bee and butterfly response patterns to climate variables were similar, we performed one-tailed unequal variance t-tests amongst each species group and their observed responses/changes.^63^ While small sample sizes, the datasets followed the assumptions required to perform an unequal variance t-test: independence of observations, no significant outliers, and normal distribution.^63^ T-tests were run for all species response categories with three or more publications available for each taxonomic group, and consistent data reporting units to follow the t-test assumption requirements. The four species responses used for t-test analyses included species emergence, richness, abundance, and distribution.

Last, to explore these responses more specifically in relation to climate variables, we performed multivariate statistics in the form of principal component analyses (PCAs) amongst publications measuring temperature change and its impact on species responses. PCAs were used to understand the multidimensionality of the datasets while minimizing the noise of data patterns. Publications with missing values for the four common response variables were not included in the PCA; consequently, this eliminated publications and their results from the final analysis. The four species responses used for the PCAs included species emergence, richness, abundance, and distribution. Both bumble bees and butterflies were grouped together in these analyses, and study areas were standardized by regional locations. To follow PCA numeric requirements, each taxon was given a code (bumble bees = 1; butterflies = 2), as were each region (North America = 1; South America = 2; Europe = 3; Asia = 4; Africa = 5; Australia = 6). Percentages provided through range distribution changes were converted into decimals. PCA analyses and plots were completed using the R packages “FactoMineR”,^64^ “factoextra”,^65^ and “corrplot”.^66^ All statistical packages, analyses, and commented codes, in addition to further PCA plot types, are available through the publication’s Mendeley data repository.^47^

## Supplemental information

Table S1. Bumble Bee/Climate Change Literature, related to Figure 1 Literature available for bumble bee and climate studies.

Table S2. Butterfly/Climate Change Literature, related to Figure 1 Literature available for butterfly and climate studies.
